# Liver Disease and Periodontal Pathogens: A Bidirectional Relationship Between Liver and Oral Microbiota

**DOI:** 10.3390/dj13110503

**Published:** 2025-10-31

**Authors:** Mario Dioguardi, Eleonora Lo Muzio, Ciro Guerra, Diego Sovereto, Enrica Laneve, Angelo Martella, Riccardo Aiuto, Daniele Garcovich, Giorgia Apollonia Caloro, Stefania Cantore, Lorenzo Lo Muzio, Andrea Ballini

**Affiliations:** 1 Department of Clinical and Experimental Medicine, University of Foggia, Via Rovelli 50, 71122 Foggia, Italy; mario.dioguardi@unifg.it (M.D.); ciro_guerra.556675@unifg.it (C.G.); diego_sovereto.546709@unifg.it (D.S.); enrica.laneve@unifg.it (E.L.); lorenzo.lomuzio@unifg.it (L.L.M.); 2 Department of Biomedical and Neuromotor Sciences, University of Bologna, Via San Vitale 59, 40125 Bologna, Italy; eleonora.lomuzio@unibo.it; 3 DataLab, Department of Engineering for Innovation, University of Salento, 73100 Lecce, Italy; angelo.martella@unisalento.it; 4 Department of Biomedical, Surgical and Dental Sciences, Istituto Stomatologico Italiano, University of Milan, 20122 Milan, Italy; riccardo.aiuto@unimi.it; 5 Department of Dentistry, Universidad Europea de Valencia, 46010 Valencia, Spain; daniele.garcovich@universidadeuropea.es; 6 Unità Operativa Nefrologia e Dialisi, Presidio Ospedaliero Scorrano, ASL (Azienda Sanitaria Locale) Lecce, Via Giuseppina Delli Ponti, 73020 Scorrano, Italy; giorgiaapollonia.caloro@asl.lecce.it; 7 Department of Precision Medicine, University of Campania Luigi Vanvitelli, 81100 Caserta, Italy; 8 Department of Life Science, Health and Health Professions, Link Campus University, 00165 Rome, Italy; a.ballini@unilink.it

**Keywords:** oral–liver axis, *Porphyromonas gingivalis*, periodontal pathogens, Metabolic Dysfunction-Associated Steatotic Liver Disease (MASLD), clinical biochemistry and molecular biology, Non-Alcoholic Fatty Liver Disease (NAFLD), randomized controlled trial, systematic review

## Abstract

**Background**: Periodontal dysbiosis contributes to liver injury through systemic inflammation, oral–gut microbial translocation, and endotoxemia. Lipopolysaccharides (LPSs) and virulence factors derived from periodontal pathogens, particularly *Porphyromonas gingivalis* (*P. gingivalis*) activate Toll-like receptor (TLR) signaling, trigger NF-κB-mediated cytokine release (e.g., TNF-α, IL-1β, IL-6), and promote oxidative stress and Kupffer cell activation within the liver. The present systematic review summarized clinical evidence supporting these mechanistic links between periodontal pathogens and hepatic outcomes, highlighting the role of microbial crosstalk in liver pathophysiology. **Methods**: A PRISMA-compliant systematic review was conducted by searching PubMed, Scopus, and the Cochrane library, as well as gray literature. Eligible study designs were observational studies and trials evaluating *P. gingivalis* and other periodontal pathogens (*Aggregatibacter actinomycetemcomitans*, *Prevotella intermedia*, and *Tannerella forsythia*) for liver phenotypes (Non-Alcoholic Fatty Liver Disease [NAFLD]/Metabolic Dysfunction-Associated Steatotic Liver Disease [MASLD], fibrosis/cirrhosis, acute alcoholic hepatitis [AAH], and Hepatocellular carcinoma [HCC]). Risk of bias was assessed using the Newcastle–Ottawa Scale adapted for cross-sectional studies (NOS-CS) for observational designs and the RoB 2 scale for single randomized controlled trials (RCTs). Due to the heterogeneity of exposures/outcomes, results were summarized narratively. **Results**: In total, twenty studies (2012–2025; ~34,000 participants) met the inclusion criteria. Population-level evidence was conflicting (no clear association between anti-*P. gingivalis* serology and NAFLD), while clinical cohorts more frequently linked periodontal exposure, particularly to *P. gingivalis*, to more advanced liver phenotypes, including fibrosis. Microbiome studies suggested stage-related changes in oral communities rather than the effect of a single pathogen, and direct translocation into ascitic fluid was not observed in decompensated cirrhosis. Signals from interventional and behavioral research (periodontal therapy; toothbrushing frequency) indicate a potential modifiability of liver indices. The overall methodological quality was moderate with substantial heterogeneity, precluding meta-analysis. **Conclusions**: Current evidence supports a biologically plausible oral–liver axis in which periodontal inflammation, often involving *P. gingivalis*, is associated with liver damage. Causality has not yet been proven; however, periodontal evaluation and treatment may represent a low-risk option in periodontitis-associated NAFLD. Well-designed, multicenter prospective studies and randomized trials with standardized periodontal and liver measurements are needed.

## 1. Introduction

Liver diseases, or hepatopathies, comprise a broad spectrum of conditions that impair the function and structure of hepatic tissues [1,2]. As a consequence, these disorders can have significant repercussions on several major systems of the human body, including the nervous and circulatory systems. Hepatic diseases frequently result from inflammatory processes known as hepatitis, which may have different etiologies: infectious origins (most commonly viral), genetic factors, autoimmune mechanisms, or exposure to certain toxins (notably alcohol).

Additional pathological entities affecting the liver include hepatic steatosis, a reversible condition characterized by the accumulation of large triglyceride vacuoles within hepatocytes. If these pathological processes persist, they may progress to cirrhosis, a state marked by the architectural distortion of hepatic tissue, hepatocyte necrosis, and subsequent replacement by fibrous tissue. Both cirrhosis and other forms of hepatopathy can ultimately lead to hepatic failure and may also increase the risk of malignancies [3].

Current management strategies emphasize prevention as the cornerstone of cirrhosis control [4]. This includes abstaining from alcohol, using specific medications with caution [5], limiting the use of non-steroidal anti-inflammatory drugs (NSAIDs) in patients with chronic hepatitis [6], and managing cardiac insufficiency pharmacologically,

A growing body of evidence highlights the bidirectional relationship between hepatopathies and the microbiota, with periodontal disease emerging as a significant actor in this interplay [7]. In fact, even adhering to a balanced diet, with an adequate sugar intake, which is one of the three factors of Keyes’ triad that contribute to tooth decay [8], and a limited salt intake (patients with hypertension), along with the use of probiotics, supports the intestinal microbial balance as well as oral health [9].

It is well established that advanced liver disease, particularly cirrhosis, negatively influences the onset and progression of periodontal disease [10]. Conversely, a high burden of bacteria responsible for periodontal disease may adversely affect liver health, increasing the risk of cirrhosis.

Mechanistically, Gram-negative bacteria and their endotoxins elicit a local host immune response, activating neutrophils, macrophages, and dendritic cells, with subsequent release of pro-inflammatory mediators such as IL-1β, IL-6, and TNF-α [11]. This sustained inflammatory response drives connective tissue breakdown and alveolar bone resorption in periodontal tissues. The ulcerated epithelium of deep periodontal pockets provides a portal of entry for microorganisms and their virulence factors into systemic circulation [12].

Notably, bacteria belonging to the “red complex,” especially *Porphyromonas gingivalis* (*P. gingivalis*), play a crucial role in the pathogenesis of periodontitis and are frequently found in large numbers within active periodontal lesions and deep periodontal pockets [13].

These specific periodontal pathogens can be easily translocated from the oral cavity to the intestine through swallowing, potentially causing substantial changes in the intestinal microbiome [14]. In fact, once translocated into the systemic circulation, bacterial products such as lipopolysaccharides (LPSs) engage Toll-like receptor 4 (TLR4) on Kupffer cells, activating NF-κB and MAPK signaling pathways [10].

This results in robust production of pro-inflammatory mediators, which amplify hepatic inflammation. Concomitantly, Kupffer cell activation generates excessive reactive oxygen species (ROS), leading to oxidative stress and mitochondrial dysfunction in hepatocytes [3]. These mitochondrial impairments compromise β-oxidation and ATP synthesis while favoring the accumulation of lipid intermediates and enhancing lipid peroxidation of cellular membranes.

The resulting hepatocellular injury not only worsens oxidative imbalance but also propagates inflammation and fibrogenic signaling, thereby accelerating the transition from simple steatosis to steatohepatitis and progressive liver disease [12].

Consequently, it has been suggested that the observed alterations in liver function are not attributable to direct inflammatory changes, but rather to modifications in the composition of the gut/oral microbiota [13]. Importantly, this relationship is bidirectional, as several studies have confirmed that advanced cirrhosis increases the risk of developing severe forms of periodontitis [13].

However, despite numerous overviews of the oral–gut–liver axis, primary clinical evidence remains fragmented across different exposures, diagnostic tools (serology, qPCR, 16S), matrices (serum, saliva, plaque), and liver phenotypes (Metabolic Dysfunction-Associated Steatotic Liver Disease—MASLD, Non-Alcoholic Fatty Liver Disease—NAFLD, viral hepatitis, alcohol-related diseases, cirrhosis/ascites, Hepatocellular Carcinoma—HCC) [15].

Recent reviews summarize plausible biological pathways [16] but do not clarify whether specific microbiological periodontal markers exist and whether associations between *P. gingivalis* and co-pathogens exist, with clearly defined liver outcomes net of confounding factors [17].

In light of this, given the lack of a study-level synthesis that systematically maps the type of exposure (*P. gingivalis* and periodontal bacteria more generally) to liver disease, the present systematic review aims to fill this gap, grouping the evidence by category of liver disease, reporting effect estimates, where available, and transparently classifying the quality of the study, seeking a possible size of the effect estimates, and assessing the risk of bias with a uniform tool.

In summary, the aim of this systematic review is to investigate the correlations existing between specific periodontal pathogens, namely *P. gingivalis*, *Aggregatibacter actinomycetemcomitans*, *Prevotella intermedia*, and *Tannerella forsythia*, in liver disease, reporting effect sizes where available, and therefore primarily providing a quantitative correlation.

## 2. Materials and Methods

### 2.1. Protocol and Registration

This systematic review was preceded by a preliminary phase that involved an in-depth analysis of the literature regarding the correlations between specific periodontal pathogens and liver disease. Based on this preliminary assessment, a decision was made to conduct a systematic review in accordance with the PRISMA (Preferred Reporting Items for Systematic Reviews and Meta-Analyses) guidelines [18]. Accordingly, the processes of literature search, study selection, and data extraction were conducted in adherence to the recommendations outlined in the Cochrane Handbook. Furthermore, the review protocol was submitted and prospectively registered on the PROSPERO platform (International Prospective Register of Systematic Reviews) under the registration number PROSPERO 2025 CRD420251126148 prior to the commencement of study selection.

### 2.2. Eligibility Criteria

All clinical and epidemiological studies investigating specific periodontal pathogens, in particular *Porphyromonas gingivalis*, *Aggregatibacter actinomycetemcomitans*, *Prevotella intermedia*, and *Tannerella forsythia*, and liver diseases were considered potentially suitable.

The PICO question formulated was as follows: In individuals diagnosed with liver diseases, does the presence or higher abundance of specific periodontal pathogens (*Porphyromonas gingivalis*, *Aggregatibacter actinomycetemcomitans*, *Prevotella intermedia*, and *Tannerella forsythia*), compared to those without these pathogens or to healthy controls, correlate with increased prevalence, severity, or progression of hepatic pathology?

✓ Population (P): Individuals (children or adults) diagnosed with liver diseases (including, but not limited to, hepatic steatosis, hepatitis, cirrhosis, or liver failure).
✓ Intervention/Exposure (I): Presence or detection of specific periodontal pathogens, namely *Porphyromonas gingivalis*, *Aggregatibacter actinomycetemcomitans*, *Prevotella intermedia*, and *Tannerella forsythia* (identified by clinical, microbiological, or molecular methods).
✓ Comparison (C): Individuals with liver diseases without detectable periodontal pathogens, or with a lower microbial burden, or, alternatively, healthy individuals without liver diseases.
✓ Outcome (O): Primary: Prevalence and/or severity of liver disease in relation to the presence or abundance of specific periodontal pathogens: Secondary: Evidence of a correlation between periodontal infection and hepatic pathology (e.g., progression, complications, inflammatory markers, or liver-related morbidity/mortality).

The inclusion criteria included cross-sectional, case–control, cohort, and interventional studies involving individuals (adults or children) with any form of liver disease, such as fatty liver disease, hepatitis, cirrhosis, or liver failure.

Studies were included that assessed the presence or abundance of these periodontal pathogens using clinical, microbiological, or molecular techniques and those reporting findings related to correlations, associations, or potential effects on liver disease.

Only articles published in English or for which a clear full-text translation was available or feasible were considered for inclusion.

Conversely, animal in vitro and in silico studies were excluded, as well as narrative reviews, editorials, commentaries, expert opinions, and case reports involving fewer than twenty patients. Studies conducted exclusively on healthy populations or individuals without liver disease, as well as those that did not specifically address the periodontal pathogens of interest, were also excluded. Articles that did not report data on the association between periodontal pathogens and liver disease were also excluded, and non-English reports were only included when a complete, accurate full-text English translation was obtainable (professional translation or validated tools with author cross-check). Studies lacking reliable full-text translation were excluded, or for which the full text or sufficient methodological details (such as abstracts or conference proceedings) was not available.

### 2.3. Sources of Information, Search Strategy and Study Selection

A pre-specified search strategy (protocol registered) was developed and applied uniformly across all information sources. Electronic searches were executed, and all records exported and de-duplicated (EndNote + manual check). Study selection (titles/abstracts, then full texts) and data extraction were performed independently in duplicate by two reviewers, with disagreements resolved by a third reviewer. Any post hoc expansion of databases/sources was documented as a protocol amendment and is reflected in the PRISMA diagram. Preliminary exclusion criteria included language restrictions: reports lacking at least an abstract in English were excluded using automated tools available within the selected databases.

The search engines and databases used included PubMed, Scopus, and the Cochrane Library. In addition, gray literature was retrieved through Google Scholar, ScienceDirect, and the DANS Archive (Data Station Life Sciences) [19]. To further reduce the risk of publication bias, the reference lists of previous reviews on liver diseases were also screened. The initial search, including the last update of identified records, was completed on 11 May 2025. A final literature update was conducted on 27 July 2025.

The search terms were selected to include the widest possible range of studies focusing on liver disease and periodontal disease.

The following search strategies were employed across the selected databases:

✓ PubMed:
  Search: (“Liver Diseases”[MeSH] OR “hepatic diseases” OR “hepatopathy” OR “hepatitis” OR “cirrhosis” OR “hepatic steatosis” OR “liver failure”) AND (“Periodontal Diseases”[MeSH] OR “periodontitis” OR “periodontal disease” OR “oral microbiome” OR “oral bacteria” OR “periodontal pathogens”) AND (“Porphyromonas gingivalis” OR “Aggregatibacter actinomycetemcomitans” OR “Prevotella intermedia” OR “Tannerella forsythia”) Sort by: Most Recent
  Query: (“Liver Diseases”[MeSH Terms] OR “hepatic diseases”[All Fields] OR “hepatopathy”[All Fields] OR “hepatitis”[All Fields] OR “cirrhosis”[All Fields] OR “hepatic steatosis”[All Fields] OR “liver failure”[All Fields]) AND (“Periodontal Diseases”[MeSH Terms] OR “periodontitis”[All Fields] OR “periodontal disease”[All Fields] OR “oral microbiome”[All Fields] OR “oral bacteria”[All Fields] OR “periodontal pathogens”[All Fields]) AND (“Porphyromonas gingivalis”[All Fields] OR “Aggregatibacter actinomycetemcomitans”[All Fields] OR “Prevotella intermedia”[All Fields] OR “Tannerella forsythia”[All Fields])
✓ Scopus:
  TITLE-ABS-KEY (“liver disease” AND “Porphyromonas”)
✓ Cochrane library:
  Search term used in Title and Abstract: “liver periodontitis”

To ensure transparency, a summary table has been included (Table 1) listing the web addresses of the databases, the date of the last search, and the number of records retrieved (excluding gray literature).

Records were exported from all sources and imported into EndNote. Duplicates were identified with EndNote’s built-in ‘Find Duplicates’ feature (default criteria) and subsequently verified and removed manually by the reviewers responsible for study selection.

Article selection was performed independently by two reviewers. Initially, they compiled lists of potentially eligible studies and subsequently included them in two separate tables, which were then compared. Potentially eligible studies were identified through title screening, while studies meeting the inclusion criteria were selected following full-text review and analysis.

Reviewer agreement was also assessed, and any discrepancies were resolved by a third reviewer.

On 1 October 2025, five additional databases not prespecified in the protocol were searched: EBSCO, Web of Science, DOAJ, Scilit, and LILACS. The EBSCO search covered the following databases: MEDLINE; MEDLINE Ultimate; APA PsycArticles; APA PsycInfo; CINAHL Ultimate; Dentistry & Oral Sciences Source; eBook Academic Collection (EBSCOhost); eBook Collection (EBSCOhost); Food Science Source; FSTA—Food Science and Technology Abstracts with Full Text; Psychology and Behavioral Sciences Collection; and SPORTDiscus with Full Text. The LILACS search covered: BBO—Dentistry and LILACS. The search terms used were “liver disease” AND “periodontitis” (Table 2).

### 2.4. Data Collection Process and Data Characteristics

The data to be included in the summary tables of the selected studies were defined during the initial development of the review protocol. As with the data assessed during the selection and screening phases, data extraction was performed independently by both reviewers and subsequently cross-checked to minimize potential inaccuracies. One reviewer then compiled the verified information into a unified table.

Any discrepancies identified during the data extraction process were initially documented and acknowledged. To address such issues, the reviewers engaged in thorough discussions to reconcile differences, clarify misunderstandings, and resolve potential errors. In cases where consensus could not be reached through discussion, unresolved issues were referred to a third reviewer. The latter carefully reviewed the disputed data and provided a final judgment on the accuracy of extraction.

To coordinate de-duplication, dual screening, data extraction and audit trails, a knowledge-base-driven workflow was implemented, with eligibility rules and decision logic encoded as structured entities. This dynamic information system approach enables traceability, controlled rule updates, and cross-process monitoring, in line with knowledge-based support frameworks for dynamic information systems [20].

For this issue, was adopted a semantically enabled, interoperable edge–cloud workflow for screening, extraction and analysis, consistent with recent e-health architecture patterns [21].

This procedure ensured that all conflicts were resolved systematically and transparently, based on the available evidence and methodologies widely used in other systematic reviews [2].

Extracted data included: first author, year of publication, study design, country of origin, number of patients included, population type mean age, sex distribution, type of liver disease, and severity or staging of liver disease presence/detection of specific periodontal pathogens.

### 2.5. Risk of Bias in Individual and Cross-Sectional Studies

Particular emphasis was placed on assessing the risk of bias, using the Newcastle–Ottawa Scale adapted for cross-sectional and observational studies and RoB 2 (risk of bias 2 tool) for RCTs (randomized controlled trial).

## 3. Results

### 3.1. Study Selection

**Figure 1:**
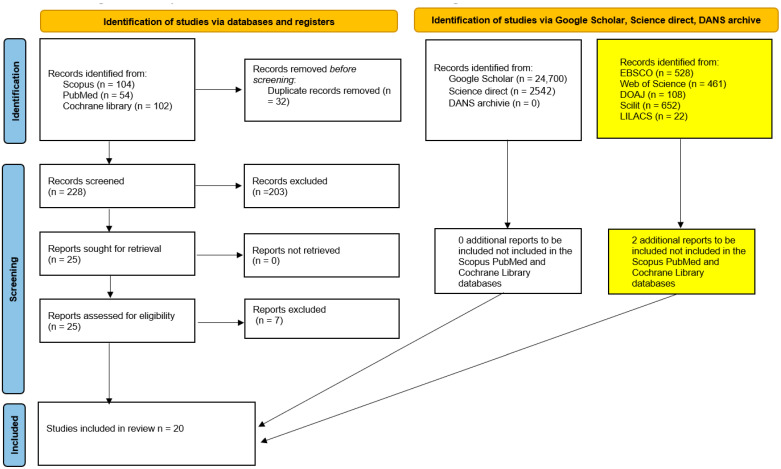
Flow chart of the study selection and inclusion process. The yellow boxes show the results of the additional search performed on 1 October 2025.

The searches conducted across SCOPUS, PubMed, and the Cochrane library yielded a total of 260 bibliographic records. After the removal of duplicates (228 records), 25 potentially eligible articles remained and were subjected to full-text screening. Of these, only 18 articles met the inclusion criteria for the systematic review; in addition to these 18 records, two further records were identified on 1 October 2025 after extending the search to five additional databases (EBSCO, Web of Science, DOAJ, Scilit, and LILACS) (Figure 1, Table 2).

Moreover, gray literature searches carried out in repositories such as DANS (Data Station Life Sciences) (https://ssh.datastations.nl/), URL (accessed on 16 July 2025) ScienceDirect, and Google Scholar, and previous systematic reviews using the keyword “liver *Porphyromonas gingivalis*”, did not allow us to identify other studies suitable for inclusion in the present systematic review.

The results of the selection and inclusion process demonstrate that there was not a sufficient number of homogeneous studies available to perform a meta-analysis of the extracted data. The complete procedure for identifying, selecting, and including studies is illustrated in the PRISMA flowchart shown in Figure 1.

During this phase, seven studies were excluded for the following main reasons:

✓ Two studies were literature reviews not explicitly identified as such in the title or abstract;
✓ Two studies were in vitro studies not explicitly identified as such in the title or abstract;
✓ One study was a research protocol of a randomized trial.
✓ One study reported data from a set of patients already included in the review, which was excluded to avoid data overlap.
✓ One study was excluded because it reported data from only five patients.

The excluded studies, along with their respective reasons for exclusion, are listed in Table 3.

The main data and results of the excluded studies are reported below.

✓ Kuraji et al., 2024 [22]. This study was excluded because it was an interventional animal model study (murine), with only a small translational component consisting of an observational human autopsy analysis. These details were not reported in the abstract or title. The findings indicated that mice with induced periodontitis exhibited a worsening of hepatic steatosis compared to controls. Furthermore, a correlation was observed between the severity of periodontitis (measured as tooth loss) and the severity of liver disease.
✓ Kuraji et al., 2023 [23]. This study was excluded because it was a narrative review (not specified in the abstract), addressing the relationships between periodontal disease, dysbiosis, and NAFLD, as well as therapeutic strategies with a focus on the microbiome.
✓ Nagao and Tsuji, 2021 [24]. This study was excluded because it was a pilot investigation involving only four cases. The main conclusion was that the elimination of HCV (Hepatitis C Virus) through direct-acting antiviral therapy in patients with hepatitis C and oral lichen planus was associated with improvements in oral lesions and a reduction in the salivary load of periodontopathogenic bacteria.
✓ Kamata et al., 2020 [25]. This publication described the protocol of a prospective, multicenter, two-arm, open-label, randomized controlled trial enrolling adult patients (20–85 years, both sexes), with NAFLD and moderate periodontitis.
✓ Wu et al., 2018 [26]. This was an in vitro study and was excluded (not stated in the title or abstract).
✓ Lê et al., 2024 [28]. This study was excluded because it was a narrative review (not stated in the title or abstract), addressing the topic of liver diseases and the oral microbiota.
✓ Emelyanov and Emelyanova, 2022 [27]. This study addressed data that had already been reported.

### 3.2. Data Characteristics

The reports included in this review comprise 20 studies published between 2012 and 2025: Tan and Xu, 2024 [29], Sato et al., 2025 [30], Matsui et al., 2024 [31], Pischke et al., 2023 [32], Pischke et al., 2023 [33], Kamata et al., 2022 [34], Sato et al., 2022 [35], Yamamoto et al., 2021 [36], Takamisawa et al., 2020 [37], Nagao and Tanigawa, 2019 [38], Zhou et al., 2018 [39], Jensen et al., 2018 [40], Nakahara et al., 2018 [41], Nagao et al., 2014 [42], Yoneda et al., 2012 [43], Emelyanov and Cherelyuk, 2022 [44], Emelyanov and Emelyanova, 2023 [45], Li et al., 2020 [46], Takuma et al., 2023 [47], and Komazaki et al., 2017 [48].

**Figure 2:**
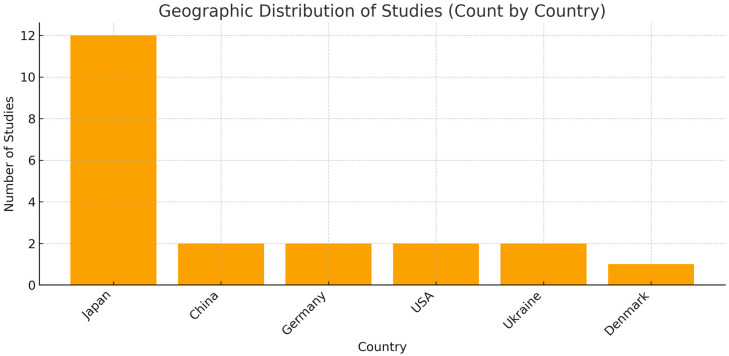
Graphical illustration of the geographical distribution of studies. The country-level count shows a strong concentration of studies in Japan (*n* = 12), whereas China (*n* = 2), Germany (*n* = 2), the USA (*n* = 2), Ukraine (*n* = 2), and Denmark (*n* = 1) contribute fewer studies. Methodological note: The chart scores studies by country; when a study involves multiple countries, it is counted under each one (e.g., Matsui 2024 is counted for both Japan and China [31]). In addition, the study labeled “China (population USA)” is counted under the USA because the sample comes from the United States. Consequently, the sum of country counts can exceed the total number of studies.

The extracted data are presented in Table 4 and Table 5 and summarize the information regarding the first author, year of publication, study design, country of origin, number of patients diagnosed with liver disease and included, sex, mean age, and Odds Ratio (OR). In addition, Figure 2 show the geographical distribution of the studies.

The number of participants varied widely across studies, ranging from 25,804 subjects in Yamamoto et al., 2021 [36] to 24 cases in Li et al., 2020 [46].

Across all included studies, 10,809 participants (30.9%) presented liver disease, whereas 24,136 (69.1%) did not suffer from the disease, yielding an overall patient/control ratio of ~1:2.23. Note that in population-based cross-sectional studies, “controls” refer to participants without the target liver condition rather than a separately recruited healthy cohort.

**Figure 3:**
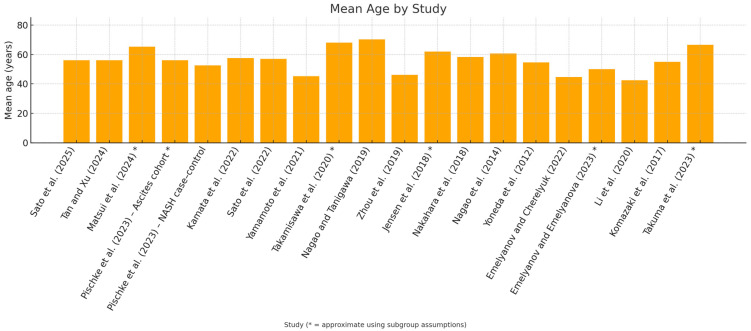
In the study-by-study [29,30,31,32,33,34,35,36,37,38,39,40,41,42,43,44,45,46,47,48], mean age graph, studies where age was approximated using subgroup assumptions have been marked with an asterisk *.

The weighted mean age was approximately ≈ 48.16 years (Figure 3).

**Figure 4:**
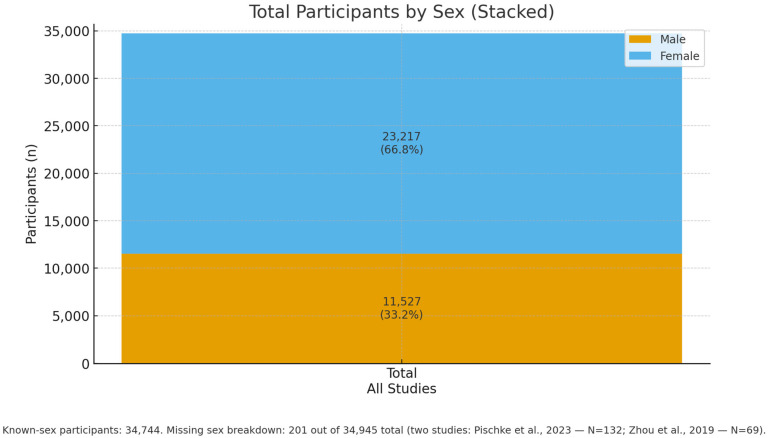
Overall sex distribution across all included studies: Males: 11,527 (33.2%), Females: 23,217 (66.8%), Participants with known sex: 34,744. M/F details are missing for 201 of 34,945 participants: the two studies Pischke et al., 2023—N = 132 (32 NASH, 100 controls) [33] and Zhou et al., 2019—N = 69 (AAH vs. healthy) [39].

In total, 23,217 female and 11,527 male patients were identified, while for approximately 201 patients, gender was not reported (two studies, Figure 4).

### 3.3. Risk of Bias

For the risk of bias, study quality was assessed using the Newcastle–Ottawa Scale adapted for cross-sectional designs (NOS-CS) as the single instrument for all observational studies (Supplemental Materials, Table S1); the single RCT (Kamata 2022), was evaluated separately with RoB 2 (Table 6), and was not included in the NOS synthesis [34]. Two reviewers independently rated the 10 NOS items (Selection S1–S5, Comparability C1–C2, Outcome/Statistics O1–O3), assigning Yes = 1 star and No/Unclear/N/A = 0, which were then mapped to overall grades (Low ≥ 7 stars; Some concerns 5–6; High ≤ 4). Full study-level judgements are reported in Table S1.

**Figure 5:**
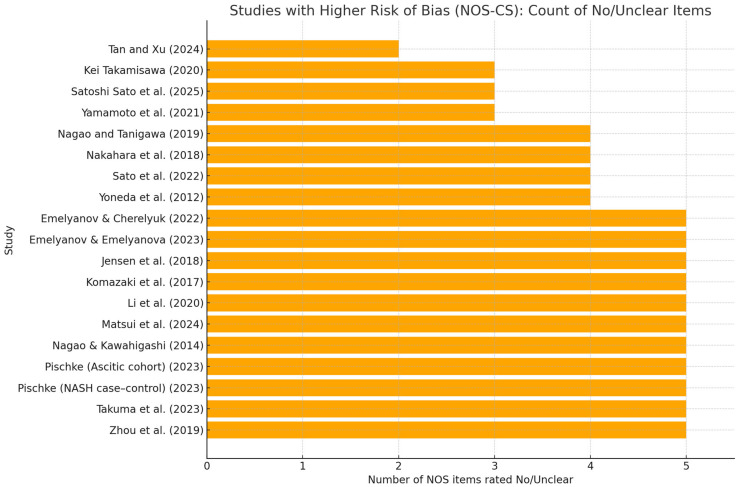
Studies with higher risk of bias (NOS-CS): count of “No/Unclear” items. Horizontal bar chart showing, for each observational study, the number of NOS-CS items rated “No” or “Unclear” out of the 10 checklist items (S1–S5, C1–C2, O1–O3). Only studies with ≥3 No/Unclear are displayed [29,30,31,32,33,35,36,37,38,39,40,41,42,43,44,45,46,47,48]; the RCT (Kamata 2022) [34] is excluded. Bars are sorted in descending order; higher bars indicate more methodological concerns, most commonly in Selection (representativeness, sample size/response) and Comparability (covariate control). This figure complements (Supplementary Materials) Table S1, which reports item-level ratings, domain stars, total stars, and the overall grade.

Overall, methodological quality was moderate, with recurrent weaknesses in the Selection and comparability domains. Many studies relied on convenience or clinic-based samples without sample-size justification or handling of non-responders (S1–S3), and covariate control was often limited to age/sex/BMI; fewer studies adjusted for additional confounders such as smoking, alcohol use, diabetes, or socioeconomic status (C2). By contrast, exposure and outcome ascertainment were generally robust: periodontal pathogens were measured using validated laboratory methods (e.g., qPCR, ELISA, 16S), and liver phenotypes were defined using objective criteria (e.g., ultrasound/CAP, elastography, biopsy, or standard biochemistry), supporting consistent Yes ratings for S4–S5 and O1–O3. More recent studies tended to report clearer diagnostic definitions and more transparent statistical approaches than earlier reports (Figure 5).

### 3.4. Study-Level Synthesis of Periodontal Exposures and Liver Outcomes

#### 3.4.1. MASLD/NAFLD

1. Tan and Xu, 2024 [29]. In a nationally representative sample of U.S. adults (NHANES III; *n* = 6330), the authors reported that there was no evidence of an association between circulating antibody responses to key periodontal pathogens (including Porphyromonas gingivalis) and ultrasound-defined NAFLD after survey-weighted adjustment; (OR 0.996; 95% CI 0.963–1.030), consistent with null effect.
2. Pischke et al., 2023 [33]. NASH severity (prospective), focusing on *P. gingivalis* and *Aggregatibacter actinomycetemcomitans* as principal periodontal pathogens, the study reported a close correlation between periodontal disease and more severe NASH, supporting the hypothesis that poor oral health may contribute to systemic inflammation and liver-disease progression (observational association; no proof of causality).
3. Sato et al., 2022 [35]. Periodontitis, particularly when associated with *P. gingivalis*, was linked to a higher risk of advanced hepatic fibrosis in NAFLD (salivary *P. gingivalis* proportion ≥ 0.01% by qPCR associated with increased liver stiffness); findings support periodontitis as a potential additional risk factor for fibrosis progression; (OR 4.05; 95% CI 1.056–15.52), but precision is low (very wide CI).
4. Yamamoto et al., 2021 [36]. Toothbrushing three times daily was associated with a significantly lower risk of NAFLD compared with less frequent brushing, independent of age, sex, BMI and lifestyle factors; the association was most apparent in individuals with obesity. The study assessed behaviors (brushing frequency) rather than specific oral bacteria.
5. Nakahara et al., 2018 [41]. Biopsy-proven NAFLD/*P. gingivalis* infection, specifically seropositivity to fimA type 4, was independently associated with advanced hepatic fibrosis in NAFLD after multivariable adjustment; (OR 2081; 95% CI 1098–3943) moderate effect, but with non-negligible uncertainty.
6. Yoneda et al., 2012 [43]. NAFLD vs. controls. *P. gingivalis* detection frequency was significantly higher in NAFLD than in non-NAFLD controls; invasive fimA genotypes were more often represented in NAFLD; (OR 2615; 95% CI 1001–6832), borderline and imprecise estimate for CI very close to unity.
7. Emelyanov and Emelyanova, 2023 [45]. NAFLD vs. healthy and cohabiting relatives. NAFLD was associated with oral dysbiosis, with increased second-order periodontopathogens (e.g., *Porphyromonas endodontalis*, *Prevotella intermedia*, *Fusobacterium nucleatum*), altered salivary parameters (reduced flow, higher viscosity, more acidic pH), and higher oral endotoxin levels.
8. Takamisawa et al. [37]. Higher anti-*P. gingivalis* IgG levels were associated with an increased risk of liver enzyme abnormalities, particularly alanine aminotransferase (ALT); effects were often sex-stratified (e.g., stronger associations in women), (OR 2.80; 95% CI 1.22–6.44); enzyme/sex-specific model, limited generalizability.
9. Sato et al., 2025 [30]. Population-based cohort (MASLD). In a large Japanese population sample, salivary 16S-based community profiles (e.g., clusters dominated by Neisseria, Streptococcus, *Fusobacterium*, *Veillonella*) were associated with MASLD phenotypes defined by CAP and cardiometabolic criteria. Pg-specific signals were not dominant; models included key behavioral and metabolic confounders. Interpretation: supports a community-level oral signal for MASLD rather than a single-pathogen effect.
10. Emelyanov & Cherelyuk, 2022 [44]. NAFLD vs. healthy (Ukraine). Using qRT-PCR for *P. gingivalis* and gingipain-K ELISA, alongside measurements of salivary physicochemical properties, NAFLD participants showed higher periodontal pathogen burden, increased oral endotoxin, and poorer salivary function, consistent with an oral–systemic inflammatory axis.
11. Komazaki et al., 2017 [48]. Oral signal centered on *Aggregatibacter actinomycetemcomitans*: higher anti-Aa IgG, as well as weakly higher anti-*Fusubacterium nucleatum*, were linked to greater visceral/total adiposity, higher fasting insulin and HOMA-IR, and a lower CT liver–spleen ratio (more steatosis). No relevant association for *P. gingivalis*. Overall, Aa-specific serology is the most consistent oral marker of a worse hepatic/metabolic phenotype in NAFLD, though evidence is correlational and unadjusted.

#### 3.4.2. Viral Hepatitis (HBV/HCV)

12. Li et al. [46]. HBV → cirrhosis → HCC (small 16S study). Across HBV, HBV cirrhosis, and HCC, shifts in salivary bacterial diversity and taxa were observed, suggesting exploratory biomarker potential (sample sizes per group were small; findings are preliminary).
13. Nagao and Tanigawa, 2019 [38]. Chronic viral hepatitis. Patients with chronic viral hepatitis exhibited greater clinical attachment loss and poorer periodontal status than comparators, indicating a heavier periodontal burden in this setting; (OR 4.08; 95% CI 1.12–15.96), limited extrapolability to a non-hepatic outcome.
14. Nagao et al., 2014 [42]. HBV/HCV liver disease. Periodontitis was associated with fibrosis progression in viral liver disease (HBV and/or HCV). Several systemic and behavioral factors (low platelet count, older age, obesity, poor oral hygiene, interferon therapy) were identified as risk factors for periodontitis.

#### 3.4.3. Alcohol-Related Disease/AAH

15. Zhou et al., 2019 [39]. Acute alcoholic hepatitis (AAH). Patients with AAH had significantly higher plasma antibodies (IgG/IgA/IgM) to *P. gingivalis* than healthy controls, especially in severe AAH and certain subgroups (e.g., women). In AAH, an active IgM response to *P. gingivalis* correlated with indices of liver injury (AST/ALT ratio, MELD), whereas long-standing alcohol exposure appeared to attenuate IgM responses.

#### 3.4.4. Fibrosis/Cirrhosis/Ascites

16. Pischke et al., 2023 [32]. Decompensated cirrhosis with ascites (prospective cohort). Using real-time PCR, *P. gingivalis* was detected in periodontal pockets in 26% and in feces in 7% of patients, but not in ascitic fluid; *Aggregatibacter* (Actinobacillus) *actinomycetemcomitans* was found in pockets in 7% and not in feces or ascites. There was no evidence of direct translocation into ascites.
17. Jensen et al., 2018 [40]. Cirrhosis with periodontitis. Among patients with cirrhosis, periodontitis was associated with a distinct subgingival microbiota compared with typical periodontitis, characterized by low abundance of classical red-complex pathogens and relative predominance of bacteria usually deemed commensals, consistent with cirrhosis-related immune dysfunction enabling altered pathogenicity.

#### 3.4.5. HCC/Malignant Progression

18. Takuma et al., 2023 [47]. Oral *P. gingivalis* and *Fusubacterium nucleatum* was found to be likely associated with the pathogenesis of NASH-HCC. Furthermore, *Fusubacterium nucleatum* was found to be negatively correlated with salivary IgA levels.
19. Matsui et al., 2024 [31]. Salivary levels of *P. gingivalis*, *Tannerella forsythia* and *Prevotella intermedia* tended to be higher in MASH-HCC than in MASH alone, but these differences did not consistently reach statistical significance. Overall salivary community structure was broadly similar between groups at the phylum and genus levels; however, *Fusobacterium nucleatum* was significantly enriched in MASH-HCC.

#### 3.4.6. Interventional Evidence

20. Kamata et al., 2022 [34]. Randomized controlled trial. Intensive periodontal therapy (scaling and root planing, SRP) in NAFLD patients with moderate periodontitis led to greater improvements in liver enzymes, steatosis indices, and endotoxin, as well as reductions in anti-*P. gingivalis* responses and improved periodontal parameters, compared with home care alone, suggesting periodontal treatment as a potential adjunctive strategy in NAFLD with periodontitis.

## 4. Discussion

Our systematic review included 20 studies that correlated liver disease with *Phorphyromonas gingivalis* and, more generally, periodontal pathogenic bacteria. These studies were primarily cross-sectional and retrospective and included a total of approximately 34,000 participants, the majority of whom were female.

Given the substantial clinical and methodological heterogeneity among studies, we refrained from meta-analyzing; the results of this narrative synthesis explicitly foreground uncertainty, highlighting wide or borderline 95% CIs, small sample sizes, and residual confounding, so that any observed associations are interpreted as hypothesis-generating rather than confirmatory (Table 5).

Overall, the data suggest a possible relationship between periodontal health and liver disease, with wide heterogeneity and predominantly observational nature. Both diseases are widely prevalent in the population in the same age groups, with a rising trend, especially for liver disease. In fact, one of the most prevalent liver conditions, showing a rising trend in recent years, with an estimated current prevalence of around 38%, is fatty liver disease.

When not attributable to chronic alcohol consumption, viral infections, or autoimmune diseases, it is termed NAFLD. In particular, NAFLD is closely linked to metabolic syndrome, obesity, type 2 diabetes mellitus, obstructive sleep apnea syndrome, hypothyroidism, hypertension, and hyperlipidemia. Mortality related to NAFLD is also increasing, largely driven by progression to non-alcoholic steatohepatitis (NASH), an inflammatory phenotype with a more aggressive course that may culminate in hepatic fibrosis and cirrhosis.

The inflammatory setting of periodontitis can propagate systemic inflammation and oxidative stress, potentially initiating or exacerbating steatosis-related hepatic inflammation. Circulating pro-inflammatory interleukins (notably IL-6 and IL-1) are frequently elevated in this context. In addition, a measurable serological indicator more reflective of periodontal infection than of generic inflammation is immunoglobulin G (IgG) directed against antigens of *P. gingivalis* and *Aggregatibacter actinomycetemcomitans*, microorganisms commonly implicated in periodontitis, with antibody titers that may remain stable for prolonged periods after infection.

Beyond systemic inflammation, direct microbial products, especially LPS and, less commonly, whole bacteria, may participate in hepatopathogenic mechanisms [49]. Members of the “red complex” (*P. gingivalis*, *Tannerella forsythia*, *Treponema denticola*) have been reported as significantly more abundant in saliva from patients with cirrhosis than in those with chronic hepatitis without cirrhosis [38].

Oral dysbiosis itself is associated with altered salivary parameters, such as reduced flow, increased viscosity, more acidic pH, and higher oral endotoxin levels [45]. Through salivary swallowing, oral taxa can reach the gut, shaping the intestinal microbiota and nutrient metabolism; the liver is functionally linked to the intestine via the portal vein and the enterohepatic circulation.

Nevertheless, in patients with decompensated chronic liver disease and ascites, periodontal pathogens have not been detected in ascitic fluid [32], despite spontaneous bacterial peritonitis being a major complication with an approximately 20% mortality risk, suggesting that indirect (inflammatory/endotoxin-mediated) routes are more plausible than frequent direct translocation.

From a preventive/therapeutic perspective, Takamisawa et al. [37] reported that higher anti-*P. gingivalis* IgG was associated with increased odds of elevated ALT (particularly in women), while Kamata et al., 2022 [34] demonstrated, in a randomized trial, that scaling and root planing (SRP) significantly reduced liver enzymes and endotoxin alongside improvements in periodontal parameters, supporting periodontal therapy as a potential adjunct for NAFLD with periodontitis. Consistently, a large retrospective cohort (~25,000 individuals) studied by Yamamoto et al., 2021 [36], found that toothbrushing three times daily was associated with lower NAFLD risk versus less frequent brushing; poor oral hygiene behaved as a risk factor for NAFLD, plausibly via chronic inflammation and oral–gut microbial pathways.

Additional evidence comes from Nakahara et al. (2018) [41], who observed that *P. gingivalis* prevalence was higher in more severe NAFLD and in advanced hepatic fibrosis, with *P. gingivalis* infection acting as an independent risk factor for fibrosis progression (NASH). In apparent contrast to “classical” pathogen-centric models, Jensen et al. [40] reported that, in cirrhosis with periodontitis, the subgingival microbiome is altered but not necessarily enriched in Socransky’s red/orange complex species [50]; rather, non-typical taxa predominate, consistent with dysbiosis secondary to cirrhosis-related immunodeficiency. In 2020, Li et al. [46] similarly documented reduced salivary bacterial diversity across chronic hepatitis B → cirrhosis → HCC, indicating dysbiosis; diversity loss may impair colonization resistance, while stage-specific changes (including increases in *Porphyromonas* spp.), suggest candidate diagnostic biomarkers for disease staging [46].

In line with Nakahara et al. (2018) [41], *P. gingivalis* infection is significantly and independently associated with fibrosis progression in NAFLD, and Yoneda et al., 2012 [43], reported a higher detection frequency of *P. gingivalis* in NAFLD than in non-NAFLD controls. *P. gingivalis* may also be relevant in alcohol-related liver disease: Zhou et al., 2018 [39], found higher plasma antibodies (IgG/IgA/IgM) to *P. gingivalis* in acute alcoholic hepatitis (AAH)—especially severe AAH—with IgM responses correlating with indices of liver injury; long-standing alcohol exposure appeared to attenuate IgM responsiveness.

It is noteworthy that in 2025, Sato et al. [35] highlighted *Veillonella*-dominated oral community profiles as potential risk signals for MASLD, with links to worsening cardiometabolic parameters and hepatic fat content [9]. This supports a community-level view of the oral–liver axis, in which specific pathogens (e.g., *P. gingivalis*) interact within broader dysbiotic networks rather than acting in isolation [35].

In the current work, it is possible to speculate a two-pathway model to contextualize the findings. First, indirect routes: periodontal inflammation and oral dysbiosis increase systemic IL-6/IL-1β and endotoxemia via oral–gut microbial crosstalk (swallowed oral taxa/LPS, gut dysbiosis, barrier dysfunction, portal delivery), activating hepatic innate immune signaling (e.g., TLR4 on Kupffer cells) and promoting steato-inflammatory and fibrotic phenotypes. Secondly, direct routes: occasional translocation of oral bacteria/DNA to hepatic compartments.

Current clinical data favor indirect mechanisms, consistent with negative ascites microbiology in decompensated cirrhosis, and align with signals of modifiability (improvement in liver enzymes/endotoxin after scaling and root planing; lower NAFLD risk with frequent toothbrushing) [51].

A recent systematic review on intestinal permeability in patients with NAFLD, conducted by De Munck [52], proposes at least two non-mutually exclusive routes by which bacterial LPS, as well as other bacterial products, can reach the liver from the oral cavity.

(1): Oral–gut portal route: periodontitis increases the swallowing of oral bacterial taxa and LPS [53]; in NAFLD/MASLD, increased intestinal permeability facilitates LPS translocation, permitting transcellular absorption into the portal vein via lipid rafts and CD36 (with a smaller contribution via chylomicrons), while chylomicron-mediated postprandial transport further augments endotoxin flux to the liver [54]. There, Kupffer cells sense LPS via TLR4, and amplify steato-inflammation and fibrogenesis.

(2): Transient bacteremia route: everyday activities (e.g., toothbrushing) can cause brief bacteremia, with hepatic clearance through the reticuloendothelial system [55].

In addition to these two principal routes, other pathways, still theoretical and not fully substantiated in patients with NAFLD, have also been considered. Beyond the portal route, an intestinal lymphatic, non-portal pathway may plausibly contribute to hepatic exposure: during fat absorption, LPS associates with chylomicrons and transits through the mesenteric lymph to the thoracic duct and the systemic circulation, ultimately reaching the liver via the hepatic artery [56].

Additionally, extracellular vesicles derived from the microbiota constitute a further nanoparticle-scale route by which LPS and bacterial nucleic acids reach the liver; emerging evidence indicates that these vesicles traverse epithelial barriers and activate Kupffer cells and hepatic stellate cells, thereby contributing to low-grade inflammation [57].

Overall, these pathways are biologically plausible, but remain supported predominantly by observational and physiological data, underscoring the need for interventional confirmation.

**Figure 6:**
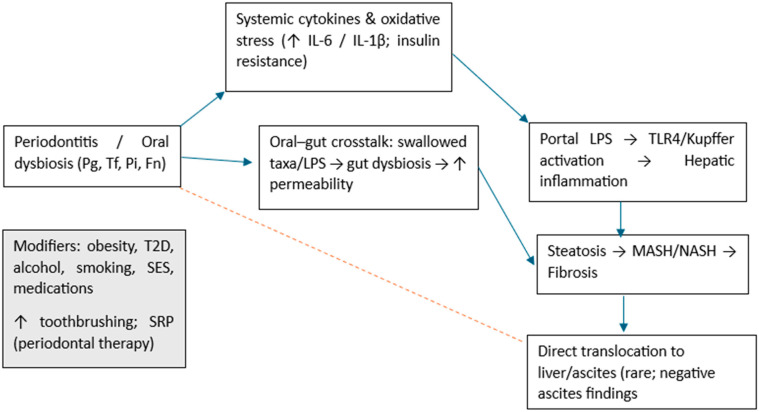
Conceptual framework for the oral–liver axis. Indirect pathways (blue) include systemic cytokines (↑ IL-6/IL-1β), oral–gut crosstalk with increased intestinal permeability, portal LPS and TLR4/Kupffer activation leading to hepatic inflammation, and progression from steatosis to MASH/NASH and fibrosis. The dotted red arrow indicates putative direct translocation to liver/ascites, which is weakly supported clinically (negative ascites findings). Modifiers (left box) include obesity, type 2 diabetes, alcohol, smoking, socioeconomic status, medications, and beneficial behaviors/interventions (frequent toothbrushing; SRP—scaling and root planning). Pg (*Porphyromonas gingivalis*); Tf (*Tannerella forsythia* ex *Bacteroides forsythus*); Pi (*Prevotella intermedia*); Fn (*Fusobacterium nucleatum*); T2D (type 2 diabetes); SES (socioeconomic status).

This framework reconciles mixed population-level serology with stronger clinic-based associations and generates testable predictions for future trials (Figure 6).

### 4.1. Limitations of the Review

The limitations of the evidence base are mostly due to the nature of the research question and the type of studies included, which were cross-sectional or retrospective, precluding temporal inference and leaving ample room for residual confounding factors (e.g., adiposity, diabetes, alcohol exposure, smoking, socioeconomic status, medications, and access to dental care).

The interpretation of associations between periodontitis and liver disease is weakened by residual confounding that is difficult to measure precisely. Obesity, particularly visceral adiposity, increases both the risk and severity of periodontitis (via systemic inflammation and dysbiosis) and hepatic progression to steatosis, MASH/NASH, and fibrosis [58]; relying solely on BMI, a basic indicator, inevitably leaves a proportion of confounding uncontrolled. Type 2 diabetes and insulin resistance (IR) worsen periodontal health and the microbiota while simultaneously accelerating liver disease [59]; measurements limited to glycemia or a binary diabetes status (yes/no) risk underestimate IR and the impact of antihyperglycemic medications, thereby leaving residual confounding. Alcohol, often under-reported, can modulate dysbiosis, intestinal permeability, and hepatic inflammation, even at moderate intakes [60].

Finally, socioeconomic status (SES), including education, income, and access to dental care, shapes oral hygiene, diet, and smoking, and is itself linked to hepatic outcomes: when SES is omitted or captured with weak proxies, part of the apparent effect of “periodontitis on liver disease”, may reflect social inequalities rather than biological mechanisms [61]. For these reasons, even adjusted estimates should be viewed as signals rather than causal proof.

Exposure assessment was heterogeneous, ranging from serology (ELISA antibodies), qPCR abundance thresholds, and 16S profiles to behavioral parameters (toothbrushing frequency) and sampling different matrices (serum, saliva, subgingival plaque). Definitions of periodontitis (clinical indices vs. parameters such as salivary occult blood) and liver endpoints (ultrasound/CAP, VCTE, MRE, biopsy, enzymes) also varied, introducing potential classification bias and limiting comparability. Clinical populations were often convenience samples from specialized settings with small sample sizes and wide confidence intervals; covariate adjustment was often limited to age/gender/BMI, with fewer studies controlling for additional confounders. The evidence spans different liver diseases (NAFLD/MASLD, fibrosis/cirrhosis, acute alcoholic hepatitis, HBV/HCV, and HCC), further increasing clinical heterogeneity. Geographically, the literature is heavily concentrated in Japan, with relatively few studies from other regions, and there is a gender imbalance among the cohorts. These characteristics, coupled with inconsistent effect metrics and selective subgroup analyses, made the synthesis inherently narrative and prevented a robust meta-analysis.

Limitations of our review methods included the following: Although duplicate screening and consensus-based data extraction was conducted, bias or subjective judgment cannot be completely ruled out, particularly when mapping heterogeneous items to NOS-CS domains and converting item-level ratings to overall scores. The single RCT was open-label and assessed separately with RoB 2, thus not impacting the observational synthesis. The conducted searches were exhaustive across major databases and gray literature sources, but we limited inclusion to full-text articles in English, so linguistic and publication bias remain possible.

Overall, these limitations justify the need for prospective, adequately powered, multicenter studies with standardized periodontal exposure (harmonized sampling/analysis and clear clinical definitions), uniform liver outcomes (agreed diagnostic thresholds), and predefined adjustment sets. Where possible, evidence from blinded outcome assessment and protocol registration studies should complement observational data to strengthen causal inference.

### 4.2. Future Directions

Future directions should prioritize multicenter randomized trials in which all potential confounders, such as obesity, smoking, SES, and concomitant pharmacotherapies, are explicitly accounted for. These studies should also examine whether achieving periodontal health precedes hepatic changes by a quantifiable interval [62], thereby demonstrating temporality (exposure preceding outcome) and a quantitative dose–response relationship, and reducing the risk of spurious interpretations due to confounding [63].

Prospective multicenter studies can strengthen the evidence by increasing their size and generalizability and, thanks to follow-up, clarifying the temporal relationship between periodontal exposure and liver phenotypes. The adoption of harmonized definitions for exposure (standardized measures and examiner calibration) and for liver outcomes (MASLD/MASH nomenclature, shared cutoffs) reduces variability between studies and misclassification. Common centralized laboratory procedures (for clinical biochemical and microbiological markers), and a prespecified set of covariates (including adiposity/diabetes/alcohol/SES), could limit confounding and improve comparability.

Finally, pre-registered protocols and analysis plans, data sharing, and uniform reporting criteria (effects with 95% CI and, if applicable, prediction interval) facilitate robust meta-analyses and a step towards causal inference [63].

## 5. Conclusions

In conclusion, although the overall picture is biologically plausible, causality has not been established. Most of the included studies are observational, and several estimates show wide confidence intervals that include values consistent with no effect. Consequently, our conclusions, while emphasizing the direction and consistency of the signals, acknowledge substantial uncertainty.

This systematic review of 20 studies (2012–2025; ~34,000 participants), supports a biologically plausible oral–liver axis linking periodontal disease to hepatic outcomes. While a large population study found no association between anti-*P. gingivalis* serology and NAFLD, clinic-based data reports relate periodontal exposure, particularly *P. gingivalis*, to more advanced liver phenotypes, although with often large CIs and potential confounding, including fibrosis in NAFLD, and to heightened immune activation in acute alcoholic hepatitis. Small 16S studies suggest stage-related shifts in salivary communities (e.g., *Fusobacterium nucleatum*, *Veillonella*), rather than a single-pathogen effect. In cirrhosis, the subgingival ecosystem appears remodeled with fewer “classical” pathogens, consistent with dysbiosis under immune dysfunction.

Mechanistically, the balance of evidence favors indirect pathways, systemic inflammation, endotoxemia and oral–gut microbial crosstalk, over frequent direct bacterial translocation; periodontal pathogens were not detected in ascitic fluid in decompensated cirrhosis. Importantly, one randomized trial shows that scaling and root planing can improve liver enzymes and endotoxin alongside periodontal parameters, and cohort data link frequent toothbrushing with lower NAFLD risk, highlighting modifiable behaviors, even if the small sample and the absence of blindness require confirmation.

Given the predominance of observational designs and heterogeneity in exposures, outcomes, and covariate control, causality cannot be inferred, and meta-analysis was not appropriate to assess this topic. Notably, only one randomized trial (open-label, *n* = 40) provides interventional data, showing short-term biochemical improvements after scaling and root planing in NAFLD with periodontitis. However, confirmatory multicenter trials are needed. Nevertheless, the overall pattern suggests that periodontal assessment and care, may represent a low-risk, potentially beneficial adjunct in patients with NAFLD or other chronic liver diseases. Future works should prioritize prospective, adequately powered, multicenter studies and well-controlled trials with standardized periodontal and hepatic measures, prespecified adjustment sets (metabolic and behavioral factors), exploration of sex-specific effects, and community-level microbial markers. Integrating oral health into hepatology pathways may yield incremental gains while definitive evidence accumulates.

## Figures and Tables

**Table 1:** This table showing the addresses of the databases records searched (* necessary to be logged into Scopus database for this search).

Research Databases or Bibliographic Databases	Web Address	Data	Records
PubMed	https://pubmed.ncbi.nlm.nih.gov/?term=%28%22Liver+Diseases%22%5BMeSH%5D+OR+%22hepatic+diseases%22+OR+%22hepatopathy%22+OR+%22hepatitis%22+OR+%22cirrhosis%22+OR+%22hepatic+steatosis%22+OR+%22liver+failure%22%29+AND+%28%22Periodontal+Diseases%22%5BMeSH%5D+OR+%22periodontitis%22+OR+%22periodontal+disease%22+OR+%22oral+microbiome%22+OR+%22oral+bacteria%22+OR+%22periodontal+pathogens%22%29+AND+%28%22Porphyromonas+gingivalis%22+OR+%22Aggregatibacter+actinomycetemcomitans%22+OR+%22Prevotella+intermedia%22+OR+%22Tannerella+forsythia%22%29&sort=date&size=200	URL (accessed on 10 July 2025)	54
	Search: (“Liver Diseases”[MeSH] OR “hepatic diseases” OR “hepatopathy” OR “hepatitis” OR “cirrhosis” OR “hepatic steatosis” OR “liver failure”) AND (“Periodontal Diseases”[MeSH] OR “periodontitis” OR “periodontal disease” OR “oral microbiome” OR “oral bacteria” OR “periodontal pathogens”) AND (“Porphyromonas gingivalis” OR “Aggregatibacter actinomycetemcomitans” OR “Prevotella intermedia” OR “Tannerella forsythia”)		
Scopus *	https://www.scopus.com/results/results.uri?st1=%22liver+disease%22+AND+%22Porphyromonas%22&st2=&s=TITLE-ABS-KEY%28%22liver+disease%22+AND+%22Porphyromonas%22%29&limit=10&origin=searchbasic&sort=plf-f&src=s&sot=b&sdt=b&sessionSearchId=221661de6b16c17aff74c1323443861b	URL (accessed on 27 July 2025)	104
	TITLE-ABS-KEY (“liver disease” AND “periodontitis”)		
Cochrane Library	https://www.cochranelibrary.com/search	10 July 2025	102 (trials)
	liver periodontitis		

**Table 2:** Search and database update.

Databases	Search	Records	Date
Ebsco	liver disease AND periodontitis	528	1 October 2025
LILACS	liver disease AND periodontitis	22	1 October 2025
DOAJ	liver disease AND periodontitis	108	1 October 2025
Scilit (MDPI)	liver disease AND periodontitis	652	1 October 2025
Web of science	liver disease AND periodontitis	461	1 October 2025

**Table 3:** Excluded studies and reasons for exclusion. Studies excluded after full-text screening with justification based on predefined eligibility criteria; RCT (randomized controlled trial).

First Author, Data, Reference	Country	Study Design	Source Database	Reason for Exclusion
Kuraji et al., 2024 [22]	Japan, USA, China	In vitro	PubMed, Web of Science, Scopus	In vitro
Kuraji et al., 2023 [23]	Japan, USA	Narrative review	PubMed, Web of Science, Scopus	Review
Nagao and Tsuji, 2021 [24]	Japan	Prospective case series, pilot study	PubMed, Web of Science, Scopus	Only four cases
Kamata et al., 2020 [25]	Japan	Protocol RCT	PubMed, Web of Science, Scopus, DOJA	Protocol
Wu et al., 2018 [26]	China	In vitro	PubMed, Web of Science, Scopus	In vitro
Emelyanov and Emelyanova, 2022 [27]	Ukraine	Cross-sectional	Scopus	Data already reported
Lê et al., 2024 [28]	France	Narrative review	Scopus	Review

**Table 4:** Characteristics of studies included in the review of liver disease and *Porphyromonas gingivalis*. N, Number; M, Male; F, Female; NAFLD, non-alcoholic fatty liver disease; MASH, Metabolic Dysfunction-Associated Steatohepatitis; HCC, Hepatocellular carcinoma; NASH, non-alcoholic steatohepatitis; SRP, scaling and root planing; C, control; TB, toothbrushing; MASLD, metabolic dysfunction-associated steatotic liver disease; ALD, Alcoholic liver disease; AAH, Acute Alcoholic Hepatitis.

First Author, Data, Reference	Country	Study Design	N of Patients Included (M/F)	Mean Age, Range, SD	Type of Liver Disease, s	Patient Liver Disease
**Sato et al., 2025** [30]	Japan	Cross-sectional	712 (312/400)	56.0 ± 14	MASLD	277
**Tan and Xu, 2024** [29]	China (population USA)	Cross-sectional	6330 (3383/2947)	56.02 ± 10.56	NAFLD	1804 (890\914)
**Matsui et al., 2024** [31]	Japan, China	Cross-sectional	41 (25/16) MASH, 19 (13/6) MASH-HCC	59 (55–70) MASH, 79 (64–82)	MASH, MASH-HCC	60
**Pischke et al., 2023** [32]	Germany	Prospective cohort study	27 (15/12)	56 (37–76) periodontitis; 59 (56–68) no periodontitis	Ascitic decompensated cirrhosis	27
**Pischke et al., 2023** [33]	Germany	Prospective cohort study	32 (16/16) NASH, 100 C	52 ± 13 periodontitis; 56 ± 15 no periodontitis	NASH	32
**Kamata et al., 2022** [34]	Japan	Randomized controlled trial	40 (18/22), 20 SRP, 20	61 ± 10 SRP, 54 ± 15	NAFLD	40
**Sato et al., 2022** [35]	Japan	Cross-sectional	164 (92/72)	57 ± 15	NAFLD	164
**Yamamoto et al., 2021** [36]	Japan	Retrospective longitudinal study	25804 (6.901/18903)	≅ 45.2 ± 13.9	NAFLD	6901
**Takamisawa et al., 2020** [37]	Japan	Cross-sectional	388 (192/196)	≅ 68.1	Subclinical liver dysfunction	388
**Nagao and Tanigawa, 2019** [38]	Japan	Cross-sectional	47 (30/17)	70.2 ± 8.8, (49–89)	Chronic hepatitis C; liver cirrhosis,	47
**Zhou et al., 2019** [39]	Usa	Cross-sectional	69	AAH: 46,1 ± 11.7; 47.0 ± 13.2 (health)	AAH, Acute Alcoholic Hepatitis	47
**Jensen et al., 2018** [40]	Denmark	Cross-sectional	21 (16/5)	62 (59–74)	Cirrhosis	21
**Nakahara et al., 2018** [41]	Japan	Retrospective	200 (94/106)	58.2 ± 13.2	NAFLD	200
**Nagao et al., 2014** [42]	Japan	Retrospective	351 (147/204)	60.62 ± 11.2, (17–87)	HCV, HBV	351
**Yoneda et al., 2012** [43]	Japan	Retrospective	210 (103/107)	52.9 ± 2.4 (health); 54.6 ± 1.2 (NAFLD)	NAFLD 48, NASH 102	150
**Emelyanov and Cherelyuk, 2022** [44]	Ukraine	Cross-sectional	146 (68/78)	44.6 ± 10.8 (NAFLD); 44.2 ± 7.2 (health)	NAFLD 126	126
**Emelyanov and Emelyanova, 2023** [45]	Ukraine	Cross-sectional	108 (43/65)	50.0 (42– 58) NAFLD)	NAFLD 44	44
**Li et al., 2020,** [46]	China	Cross-sectional	24 (12/12)	≅42.5 ± 3.6	Hepatitis B, hepatitis B cirrhosis; liver cancer	18
**Takuma et al., 2023** [47]	Japan	Cross-sectional pilot study	60 (36/24)—NASH 40 (22/18), NASH-HCC 20 (14/6) (M/F)	NASH 60.5 (55–70), NASH-HCC 78.5 (65–81.8)	NASH 40, NASH-HCC 20	60
**Komazaki et al., 2017** [48]	Japan	Cross-sectional	52 (27/25)	55 ± 13.8	NAFLD	52

**Table 5:** Odds ratios (ORs) for *P. gingivalis* and liver-related outcomes: Notes: ORs were reported as adjusted where available and correspond to the specific exposure/outcome definition in each paper. Nagao & Tanigawa (2019) [38] report an oral microbiological endpoint (high red complex) rather than hepatic disease; Takamisawa (2020) [37] presents enzyme-specific, sex-stratified models—here we report the clearly stated association for elevated ALT in women. Exploratory pooling (Sato 2022 [35] + Yoneda 2012 [43]). A pooled log (ORs) was performed using inverse-variance weighting; with k = 2, the random-effects model collapses to fixed effects (τ^2^ ≈ 0). The pooled estimate was OR = 3.03 (95% CI 1.39–6.62; I^2^ = 0%; k = 2). Study weights were Yoneda ≈ 66% and Sato ≈ 34%. Interpretation: this is a positive signal but, given the heterogeneous hepatic outcomes, it should be considered purely exploratory and interpreted with great caution. NAFLD, non-alcoholic fatty liver disease; MASH, Metabolic Dysfunction-Associated Steatohepatitis; HCC, Hepatocellular carcinoma; NASH, non-alcoholic steatohepatitis; C, control; MASLD, Metabolic Dysfunction-Associated Steatotic Liver Disease; ALD, Alcoholic liver disease; AAH, Acute Alcoholic Hepatitis; ALT, Alanine Aminotransferase; BMI, Body Mass Index; ELISA, Enzyme-Linked Immunosorbent Assay; MRE, Magnetic Resonance Elastography; PCR, polymerase chain reaction; CI, confidence interval.

Study (Year)	Outcome (Definition)	Exposure (Definition)	OR 95% CI	*p* Value	Notes
**Yoneda et al., 2012** [43]	NAFLD (biopsy-proven) vs. controls	Salivary *P. gingivalis* (PCR/fimA)	2.615 (1.001–6.832)	0.049	Adjusted for age, diabetes, and BMI.
**Nagao and Tanigawa, 2019** [38]	High oral ‘red complex’ burden (microbiological endpoint)	*P. gingivalis* fimA II genotype	4.08 (1.12–15.96)	0.0336	Endpoint is oral microbiological load; cirrhosis is an independent covariate in the model.
**Nakahara et al., 2018** [41]	Advanced fibrosis in NAFLD	Serum anti-*P. gingivalis* fimA type 4 positivity	2.081 (1.098–3.943)	0.025	Adjusted association reported.
**Sato et al., 2022** [35]	Advanced fibrosis in NAFLD (MRE ≥ 3.4 kPa)	Salivary *P. gingivalis* ≥ 0.01% (PCR)	4.05 (1.056–15.52)	0.04	Adjusted association present.
**Tan and Xu, 2024** [29]	NAFLD (ultrasound, NHANES III)	Mixed anti-*P. gingivalis* antibodies (serum)	0.996 (0.963–1.030)	0.81	No association after survey-weighted adjustment.
**Takamisawa et al. (2020)** [37]	Elevated ALT (females), T3 vs. T1 anti-*P. gingivalis* IgG	ELISA anti-*P. gingivalis* IgG (tertiles)	2.80 (1.22–6.44)	<0.05	Outcome-specific; study reports enzyme-specific, sex-stratified models (no single composite OR).

**Table 6:** Risk of bias (RoB 2)—randomized controlled trial study: Kamata et al., 2022 (Japan)—RCT, SRP (scaling and root planning) vs. toothbrushing (control), *n* = 40 [34]. Registration: UMIN000022079. Primary endpoint: Change in ALT at 12 weeks (ITT, Intention-to-Treat). The trial was open-label; however, outcomes were primarily objective laboratory or imaging measures. The main source of potential bias relates to deviations from intended interventions in the absence of blinding. Overall RoB 2 judgment: Some concerns.

Domain (RoB 2)	Judgment	Rationale (Concise)
1. Bias arising from the randomization process	Low risk	Computer-generated sequence with concealed allocation via a central system; baseline characteristics balanced; no evidence of compromised randomization.
2. Bias due to deviations from intended interventions	Some concerns	Open-label behavioral intervention (SRP vs. toothbrushing) could influence adherence or co-interventions; primary analysis by ITT, but lack of blinding may introduce performance bias.
3. Bias due to missing outcome data	Low risk	No substantial differential attrition for the 12-week primary endpoint; outcome data largely complete in both arms.
4. Bias in measurement of the outcome	Low risk	Outcomes largely objective (biochemistry, elastography/imaging) with standardized assays; lack of blinding unlikely to affect measurement.
5. Bias in selection of the reported result	Low risk	Registered protocol with prespecified primary endpoint and analysis plan; reported estimates and CIs consistent with protocol—no indication of selective reporting.

## Data Availability

No new data were created or analyzed in this study. Data sharing is not applicable to this article.

## Supplementary Materials

The following supporting information can be downloaded at https://www.mdpi.com/article/10.3390/dj13110503/s1. Table S1: Newcastle–Ottawa Scale adapted for cross-sectional designs (NOS-CS) risk of bias table.
