# Supplementary material for: Liver Disease and Periodontal Pathogens: A Bidirectional Relationship Between Liver and Oral Microbiota
Source: Dent J (Basel). 2025 Oct 31;13(11):503. doi: 10.3390/dj13110503 (PMC12651693; doi:10.3390/dj13110503)
Supplement: Supplementary file 1 [file dentistry-13-00503-s001.zip › dentistry-3865446-supplementary.pdf]

Table S1. Risk of bias assessment (NOS-CS) of the included studies. The methodological quality of the observational studies was evaluated using the Newcastle–Ottawa Scale adapted for cross-sectional studies (S1–S5, C1–C2, O1–O3). For each item, judgments were reported as Yes/No/Unclear (Yes = 1 star), with stars assigned to each domain (Selection, Comparability, Outcome/Statistics), along with the total score and the overall quality rating (Low ≥ 7; Some concerns = 5–6; High ≤ 4). The randomized controlled trial (Kamata 2022) was recorded as N/A and did not contribute to the scoring.

Study	Year	Country	Design	Tool	S1 Representativeness of the sample	S2 Sample size justification/power	S3 Non-respondents (response rate/analysis)	S4 Ascertainment of exposure (measurement quality)	S5 Ascertainment of outcome (objective criteria)	C1 Comparability: adjusted for age/sex/BMI (or equivalents)	C2 Comparability: additional factors (smoking, diabetes, alcohol, etc.)	O1 Outcome assessment valid & reliable	O2 Appropriate statistical analysis	O3 Effect size with precision (CI) or p-values	Sel stars	Comp stars	Out stars	Total	Grade	Notes/Justification
Tan and Xu (2024) [29]	2024	USA (NHANES III)	Cross-sectional	NOS-CS	Yes	Unclear	Unclear	Yes	Yes	Yes	Yes	Yes	Yes	Yes	3	2	3	8	Low	NHANES III; ultrasound NAFLD; standardized serum IgG; survey-weighted models.
Takamisawa et al. (2020) [37]	2020	Japan	Cross-sectional	NOS-CS	No	No	No	Yes	Yes	Yes	Yes	Yes	Yes	Yes	2	2	3	7	Low	Hospital outpatients (PROST); ELISA IgG Pg; liver enzymes; multivariable models.
Nagao and Tanigawa (2019) [38]	2019	Japan	Cross-sectional	NOS-CS	No	No	No	Yes	Yes	Yes	Unclear	Yes	Yes	Yes	2	1	3	6	Some concerns	Specialist clinics; saliva PCR; liver status imaging/biopsy/FIB-4; multivariable logistic regression.
Matsui et al. (2024) [31]	2024	Japan; China	Cross-sectional	NOS-CS	No	No	Unclear	Yes	Yes	Unclear	Unclear	Yes	Yes	Yes	2	0	3	5	Some concerns	Pilot; 16S rRNA; MASH vs MASH-HCC; Bayesian network; limited covariate adjustment.
Pischke (Ascitic cohort) (2023) [32]	2023	Germany	Prospective cohort	NOS-CS	No	No	No	Yes	Yes	No	No	Yes	Yes	Yes	2	0	3	5	Some concerns	Prospective ascites cohort; PCR pathogens; mainly univariate/KM.
Pischke (NASH case–control) (2023) [33]	2023	Germany	Case–control	NOS-CS	No	No	Unclear	Yes	Yes	No	No	Yes	Yes	Yes	2	0	3	5	Some concerns	32 NASH vs 100 controls; periodontal indices; hepatic parameters; limited adjustment.
Emelyanov & Emelyanova (2022) [45]	2022	Ukraine	Cross-sectional	NOS-CS	No	No	Unclear	Yes	Yes	Unclear	Unclear	Yes	Yes	Yes	2	0	3	5	Some concerns	NAFLD vs healthy; qPCR Pg + gingipain K ELISA; standard NAFLD diagnostics.
Jensen et al. (2018) [40]	2018	Denmark	Cross-sectional (single-arm)	NOS-CS	Unclear	No	Unclear	Yes	Yes	No	No	Yes	Yes	Yes	2	0	3	5	Some concerns	21 cirrhosis+periodontitis; standardized exam; subgingival 16S; PCoA/PERMANOVA.

Kamata et al. (2022) [34]	2022	Japan	Randomized controlled trial	N/A (RCT)															N/A	
Emelyanov & Cherelyuk (2022) [44]	2022	Ukraine	Cross-sectional	NOS-CS	No	No	Unclear	Yes	Yes	Unclear	Unclear	Yes	Yes	Yes	2	0	3	5	Some concerns	NAFLD (n=126) vs healthy (n=20); qRT-PCR + gingipain K ELISA; endotoxin & saliva measures.
Zhou et al. (2019) [39]	2019	USA	Cross-sectional (AAH vs healthy)	NOS-CS	No	No	Unclear	Yes	Yes	No	No	Yes	Yes	Yes	2	0	3	5	Some concerns	AAH (n=47) vs healthy (n=22); whole-cell ELISA antibodies to Pg; ANOVA/linear models.
Yoneda et al. (2012) [43]	2012	Japan	Retrospective cross-sectional	NOS-CS	No	No	No	Yes	Yes	Yes	No	Yes	Yes	Yes	2	1	3	6	Some concerns	NAFLD biopsy (n=150) vs controls (n=60); saliva PCR Pg/fimA; multivariable regression (age, DM, BMI).
Nagao & Kawahigashi (2014) [38]	2014	Japan	Retrospective cross-sectional (HBV/HCV)	NOS-CS	No	No	Unclear	Yes	Yes	Unclear	Unclear	Yes	Yes	Yes	2	0	3	5	Some concerns	— HBV/HCV (n=351) salivary occult blood; sub-study (n=28) Pg fimA PCR-Invader
Sato et al. (2022) [35]	2022	Japan	Cross-sectional	NOS-CS	No	Yes	Unclear	Yes	Yes	Unclear	Unclear	Yes	Yes	Yes	3	0	3	6	Some concerns	NAFLD clinic (n=164); saliva PCR Pg ratio threshold; outcomes by MRE/VCTE/MRI-PDFF; logistic regression.
Sato et al. (2025) [30]	2025	Japan	Cross-sectional (population-based)	NOS-CS	No	No	Unclear	Yes	Yes	Yes	Yes	Yes	Yes	Yes	2	2	3	7	Low	Population-based; 16S saliva; MASLD by CAP + cardio-metabolic criteria; adjusted models.
Yamamoto et al. (2021) [36]	2021	Japan	Retrospective longitudinal	NOS-CS	No	No	Unclear	Yes	Yes	Yes	Yes	Yes	Yes	Yes	2	2	3	7	Low	Retrospective longitudinal cohort; toothbrushing frequency; NAFLD by ultrasound; multivariable models.
Nakahara et al. (2018) [41]	2018	Japan	Retrospective (biopsy-proven NAFLD)	NOS-CS	No	No	Unclear	Yes	Yes	Yes	Unclear	Yes	Yes	Yes	2	1	3	6	Some concerns	Biopsy-proven NAFLD (n=200); serum IgG anti-Pg fimA types; multivariate analysis (fimA type 4).
Li et al. (2020) [46]	2020	China	Cross-sectional (HBV/HBV cirrhosis/HCC vs healthy)	NOS-CS	No	No	Unclear	Yes	Yes	No	No	Yes	Yes	Yes	2	0	3	5	Some concerns	16S saliva; HBV (n=6), HBV cirrhosis (n=6), liver cancer (n=6) vs healthy (n=6); diversity & differential taxa; limited covariate adjustment.

Komazaki et al. (2017) [48]	2017	Japan	Cross-sectional (NAFLD clinic)	NOS-CS	No	No	No	Yes	Yes	No	No	Yes	Yes	Yes	2	0	3	5	Some concerns	Single-centre NAFLD sample; no power calculation or non-responder analysis. Exposure measured with validated IgG ELISAs (Aa/Pg/Fn); hepatic outcomes were objective (CT liver–spleen ratio, liver enzymes). Analyses were predominantly correlational, with no adjustment for age, sex, BMI or other confounders; p values were reported.
Takuma et al. (2023) [47]	2023	Japan	Cross-sectional pilot (NASH vs NASH-HCC)	NOS-CS	No	No	No	Yes	Yes	No	No	Yes	Yes	Yes	2	0	3	5	Some concerns	Pilot, single-centre study; no power calculation and no non-responder analysis. Exposure measured via IgG against P. gingivalis/F. nucleatum and salivary abundance; outcomes were standard clinical categories (NASH/NASH-HCC). Comparisons were non-parametric and χ^2, with no adjusted models; p values were reported
